# Editorial: Direct modulation of ion channels by G-proteins

**DOI:** 10.3389/fphys.2024.1465766

**Published:** 2024-08-09

**Authors:** Daniel Yakubovich

**Affiliations:** ^1^ Department of Neonatology, Sanz Medical Center-Laniado Hospital, Netanya, Israel; ^2^ Adelson School of Medicine, Ariel University, Ariel, Israel

**Keywords:** ion channel, G-protein, signal transduction, receptor, effector

Heterotrimeric G proteins are central mediators of intracellular signaling and are involved in control of many processes such as heart rate and blood pressure modulation, hormone secretion and release, renal function and cognitive processes. Activation of heterotrimeric G proteins by G protein-coupled 7 transmembrane spanning receptors (GPCRs) is a complex process, the subject of an intensive research that contributes to growing a list of drugs, such as β-adrenergic receptor blockers, opioids, anti-histamine drugs, dopamine receptors agonists and antagonists, etc. These drugs are utilized for the treatment of common pathologies, such as hypertension, pain, allergies, asthma, motor diseases and psychiatric conditions (Liu et al., 2024).

The list of heterotrimeric G-protein effectors is ever extending, from adenyl cyclase in the early years of G-protein research to potassium channels (GIRK – G-activated potassium channels family) (Luo et al., 2022), voltage dependent N-type Ca2+ channels (Jurkovicova-Tarabova and Lacinova, 2019), phospholipases (Ubeysinghe et al., 2023), GRK (G-protein activated receptor kinases family) (Zhang et al., 2024), KCNQ channels, beta-arrestins, etc., (Smrcka and Fisher, 2019). The current article Research Topic focuses on direct modulation of ion channels by G-proteins. Three review articles summarize research data about important G-protein effectors, in particular GIRK channels, KCNQ channels and TRP channels (Kang et al.; Nguyen et al.; Stott and Greenwood). Two additional original research articles describe the TRPC1-TRPC5 response to G proteins and modulation of stargazin (CaVγ2 subunit) expression by a cAMP dependent mechanism (Muñoz-Herrera et al.).

The mechanism of heterotrimeric G-proteins activation is still subject of intense scientific research. Since the description of the classical activation cascade involving GDP/GTP exchange (Selinger-Cassel cycle) and the subsequent dissociation of GαGTP and Gβγ, with termination of G-protein cycle accomplished by the Gα-mediated GTP hydrolysis and subunit re-association (Gilman, 1995), quite a lot of information was obtained utilizing ever extending research methods such as utilization of heterologous expression systems, Forster Resonance Energy Transfer (FRET) and single molecule monitoring techniques. Previously G-protein dissociation was assumed obligatory for interaction with effectors. At least for several signal conduction cascades rearrangement of G-protein subunits without physical separation of Gα from Gβγ is now considered (Lambert, 2008). Furthermore, the idea of multi-protein complexes comprising the GPCR, G-protein and effector, and also other modulatory molecules, is gaining support, explaining in some cases the high speed and high fidelity of signal transduction cascades which are higher than expected from diffusion-limited amplification models described previously (Doupnik, 2008). Moreover, while Gβγ was previously considered as only signal terminating subunit, nowadays quite a few effectors directly activated by an interaction with Gβγ have been described (Smrcka and Fisher, 2019). Additionally, some of the effectors are modulated directly by both branches of G-protein signaling cascade, i.e., both Gα and Gβγ, such as in the case of some phospholipases and GIRK channels).

G-protein cycle is not an isolated signal transduction pathway. A plethora of modulatory molecule such as RGS [regulator of G-protein signaling (Chidiac, 2016)], AGS [activator of G-protein signaling (Blumer and Lanier, 2014)] and even monovalent cations [such as Na^+^ (Friedman et al., 2020)] influence kinetics and amplitude of G-protein dependent signaling. Furthermore, GPCR-G-protein interaction was shown to be at least partially voltage dependent (Vickery et al., 2016; Tauber and Ben-Chaim, 2024). Moreover, elaboration of crystal structures of protein complexes, which incorporate G-proteins and other molecules [GPCRs, effectors, modulatory molecules (Weis and Kobilka, 2018)], sheds additional light on structure-functional correlations of G-proteins activity.

In addition to the vast amount of knowledge about normal function of G-proteins, there is a growing field of research oriented to study the involvement of G-proteins in disease. In particular, various mutations in G-protein molecules have been shown to influence endocrine function [McCune-Albright syndrome (Nicolaides et al., 2023)], epilepsy and neuro-development [GNB1 encephalopathy (Nasvytis et al., 2024)]. Furthermore, substantial contribution was shown for GIRK channels in the development of addictions and pathophysiology of cardiac arrhythmias such as atrial fibrillation (Mitrokhin et al., 2024).

To summarize, G-protein research is an extending field of enquiry of constantly growing physiological and pharmacological importance. Four scientists (Alfred G. Gilman, Martin Rodbell, Brian Kobilka, and Robert Lefkowitz) received Noble Prize for substantial contribution to our knowledge in this field and there is still a lot to continue the research in it.
